# Cyclosporine-A-Induced Intracranial Thrombotic Complications: Systematic Review and Cases Report

**DOI:** 10.3389/fneur.2020.563037

**Published:** 2021-02-11

**Authors:** Si-ying Song, Zhong-ao Wang, Yu-chuan Ding, Xun-ming Ji, Ran Meng

**Affiliations:** ^1^Department of Neurology, Xuanwu Hospital, Capital Medical University, Beijing, China; ^2^Advanced Center of Stroke, Beijing Institute for Brain Disorders, Beijing, China; ^3^Department of China-America Institute of Neuroscience, Xuanwu Hospital, Capital Medical University, Beijing, China; ^4^Department of Neurosurgery, Wayne State University School of Medicine, Detroit, MI, United States

**Keywords:** cyclosporine-A, cerebral venous sinus thrombosis, cerebral arterial infarction, case report, systematic review

## Abstract

This study reported two cases of intracranial thrombotic events of aplastic anemia (AA) under therapy with cyclosporine-A (CsA) and reviewed both drug-induced cerebral venous thrombosis (CVT) and CsA-related thrombotic events systematically. We searched PubMed Central (PMC) and EMBASE up to Sep 2019 for publications on drug-induced CVT and Cs-A-induced thrombotic events. Medical subject headings and Emtree headings were used with the following keywords: “cyclosporine-A” and “cerebral venous thrombosis OR cerebral vein thrombosis” and “stroke OR Brain Ischemia OR Brain Infarction OR cerebral infarction OR intracerebral hemorrhage OR intracranial hemorrhage.” We found that CsA might be a significant risk factor in inducing not only CVT but also cerebral arterial thrombosis in patients with AA.

## Background

Cyclosporine-A (CsA) is widely used as an immunosuppressive agent in organ transplantation (1–3), ulcerative colitis (UC) (4–6), and aplastic anemia (AA) (7). Most commonly, the high incidences of thromboembolic complications in the renal vascular system were found in patients with CsA use after kidney transplantation (8, 9), which might be due to acute and chronic nephrotoxicity of CsA. However, thrombotic complications in other organs secondary to CsA use are not fully analyzed in the clinical settings (10). In particular, cases of CsA-induced intracranial thrombotic complications in patients with AA were rather rare (7). Herein, we presented two cases of AA with CsA-related intracranial thrombotic events, involved in cerebral venous sinuses and cerebral arteries, respectively. Besides, we conducted a systematic literature review of CsA-related thrombotic events to give more clinical references to physicians in this field.

Moreover, it is well-known that oral contraceptive (OCP) use is regarded as the iatrogenic risk factor inducing cerebral venous thrombosis (CVT). However, there is by far no review on if any other medications that could also cause CVT. Therefore, inspired by our case of CsA-induced CVT, we further comprehensively reviewed drug-induced CVT.

## Case Presentation

### Case 1

A 15-year-old female with a 4-year history of AA with treatment of CsA (50 mg, bid) complained of an intermittently severe headache on her left frontoparietal areas for 8 months. Her headache could initially attenuate after intravenous injection of mannitol (125 ml, q8h) for 7 days. However, her headache was recurrent and even became aggressively severe with nausea and projectile vomiting 20 days ago, which could no longer be relieved by the former treatment of mannitol. Physical examination revealed a body temperature of 36.4°C, blood pressure of 105/85 mmHg, heart rate of 78/min, and respiratory rate of 20/min. No abnormal finding was found in the neurological examination. Fundoscopy showed stage V papilledema measured by the Frisén scale (Supplementary Figure 1).

**Figure 1:**
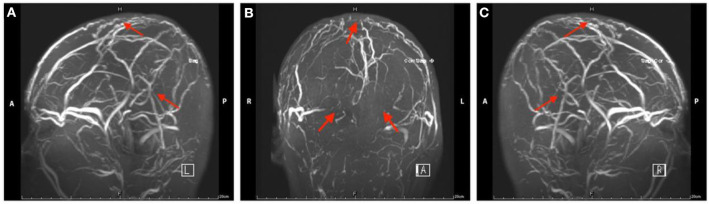
Magnetic resonance venography images of head in Case 1. The red arrow indicates the focal stenosis of cerebral venous sinus and venous collateral circulation.

**Figure 2:**
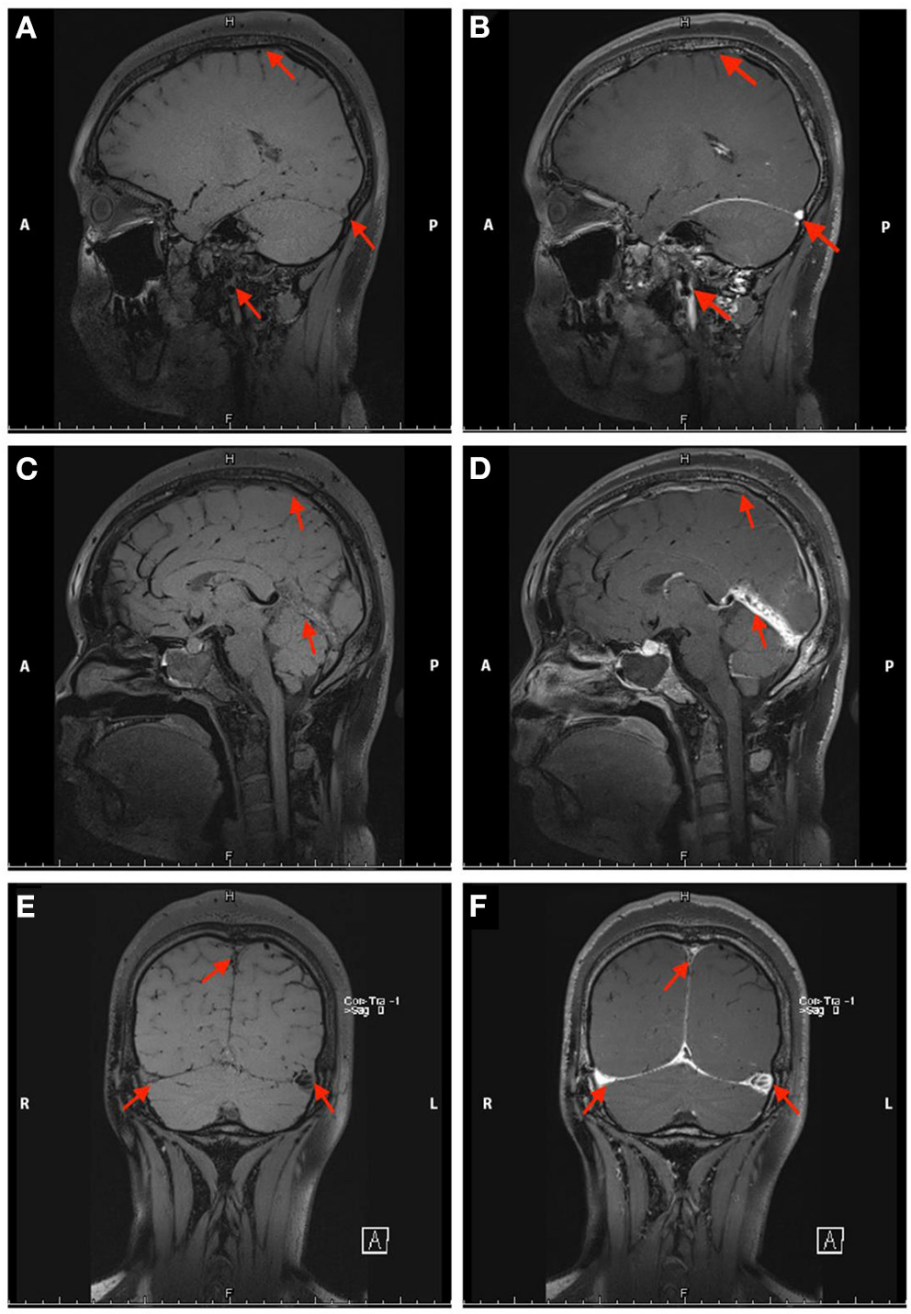
Non-contrast enhanced **(A,C,E)** and contrast-enhanced **(B,D,F)** black-blood thrombus images of the head in Case 1. The red arrow indicates the focal stenosis of the internal jugular vein and cerebral vein sinus.

Her complete blood cell (CBC) test indicated moderate normocytic normochromic anemia and a decreased platelet level due to her primary disease. The serum iron test was normal, which further excluded the differential diagnosis of iron deficiency anemia. Baseline levels of inflammatory biomarkers, including C-reactive protein (CRP) (37.2 mg/L, normal 1.0–8.0 mg/L), high-sensitivity CRP (hs-CRP) (25.75 mg/L, normal 0.0–3.0 mg/L), and interleukin 6 (IL-6) (19.6 pg/ml, normal 0.0010–7.0 pg/ml) were all above the upper normal limits (Supplementary Table 1), which suggested acute inflammatory reaction secondary to the primary disease. An increased level of D-dimer (2.47 μg/ml, normal range 0.01–0.5 μg/ml) and fibrinogen (4.21 g/L, normal range 2.0–4.0 g/L) remained over the upper limit of the normal range for several days after admission, suggesting the formation of thrombosis at acute stage (Supplementary Table 1). Serum neuron-specific enolase (NSE) level at admission was 51.52 ng/ml (normal range 0.0–17.0 ng/ml). The elevated NSE was related to damage to both neurons and the blood–brain barrier (BBB). Investigation for vasculitis [antinuclear antibody (ANA), antineutrophil cytoplasmic antibody (ANCA), and antiphospholipid antibody (APLA)] was negative. The cerebrospinal fluid (CSF) profile revealed a slightly increased white blood cell (WBC) count (2 × 10^6^/L), and lumbar puncture opening pressure (LPOP) was over 330 mm H_2_O. Contrast-enhanced magnetic resonance venography (CE-MRV) (Figure 1) and high-resolution MRI with black-blood thrombus image (MRBTI) of the brain (Figure 2) demonstrated subacute thrombosis in the superior sagittal sinus (SSS), straight sinus, right transverse sinus (TS), right sigmoid sinus (SS), and proximal part of right internal jugular vein (IJV). Moreover, no parenchymal lesion was found in MRBTI. The confirmed diagnosis of subacute CVT in multiple sites was made based on imaging findings, with involvement in cerebral venous sinuses and IJV. The CVT-induced cerebrospinal venous insufficiency could cause disturbance of CSF circulation, further leading to intracranial hypertension and related symptoms, such as severe headaches and projectile vomiting. However, the etiology of CVT development was hard to be explained in this case due to lacking common risk factors like other female CVT patients, such as obesity, pregnancy, or long-term OCP use. Moreover, no positive result was found in the workup of thrombophilia, including protein S (PS), protein C (PC), antithrombin-III (AT-III), Factor VII/VIII deficiency, or Factor V Leiden mutation. Then, we closely monitored her blood cell counts on an everyday basis. Her hypercoagulable state induced by moderate anemia secondary to AA and probable adverse effect of CsA on damaging venous vessel walls raised our attention. The procoagulant effect of the two factors might potentiate the formation of CVT.

Intravenous injection of mannitol (125 mL, q8h) was continued after the admission. Subcutaneous injection enoxaparin sodium (0.6 ml, qd) was started when the diagnosis of CVT was confirmed and usage of CsA was suspended after consultation with the department of hematology. The usage of enoxaparin sodium was then bridged to rivaroxaban (20 mg, qd) when she was discharged. Outpatient follow-up after 6 months of standard anticoagulation was evaluated by the Patients' Global Impression of Change (PGIC) scale. The patient reported a definite improvement of her symptoms (PGIC score = 6) and was transferred to the department of hematology to further treat AA.

### Case 2

A 34-year-old male with a 1-year treatment of CsA (50 mg, bid) for AA presented with right homonymous hemianopia for 20 days, accompanied by dizziness and right-hand numbness. There was no history of nausea and vomiting, motor or sensory symptoms in the limbs, facial bulbar symptoms, sphincter incontinence, and loss of consciousness or seizures. He denied a family history of blood clotting disorders. Physical examination showed his body temperature was 36.9°C, blood pressure was 130/84 mmHg, heart rate was 72 beats/min, and respiratory rate was 18 beats/min. Neurological examination revealed no positive findings.

**Figure 3:**
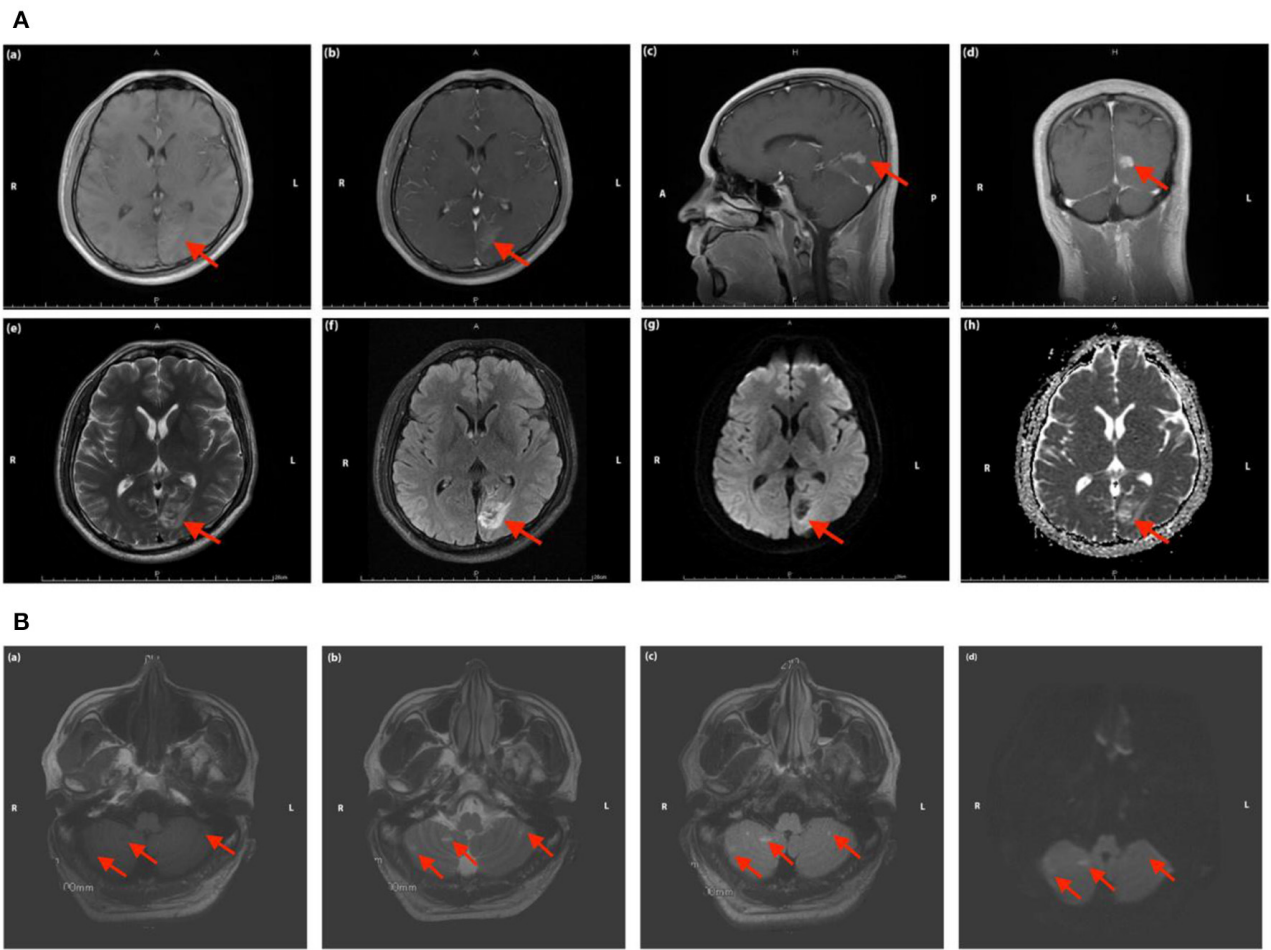
**(A)** Magnetic resonance images of the head in Case 2. The red arrow indicates the focal ischemic infarction in left occipital lobe [**(a)** T1 sequence; **(b–d)** T1 sequence with contrast-enhancing; **(e)** T2 sequence; **(f)** T2 FLAIR sequence; **(g)** DWI sequence; **(h)** ADC sequence]. **(B)** Magnetic resonance images of the head in Case 2. The red arrow indicates the focal ischemic infarction in cerebellum [**(a)** T1 sequence; **(b)** T2 sequence; **(c)** T2 FLAIR sequence; **(d)** DWI sequence].

**Figure 4:**
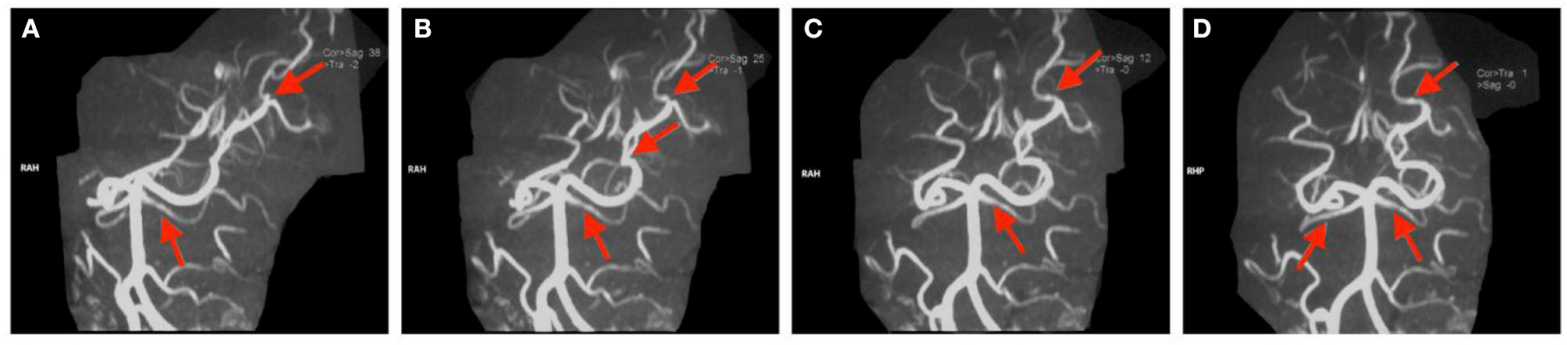
**(A–D)** Magnetic resonance arthrography images of the head in Case 2. The red arrow indicates partial filling defects.

**Figure 5:**
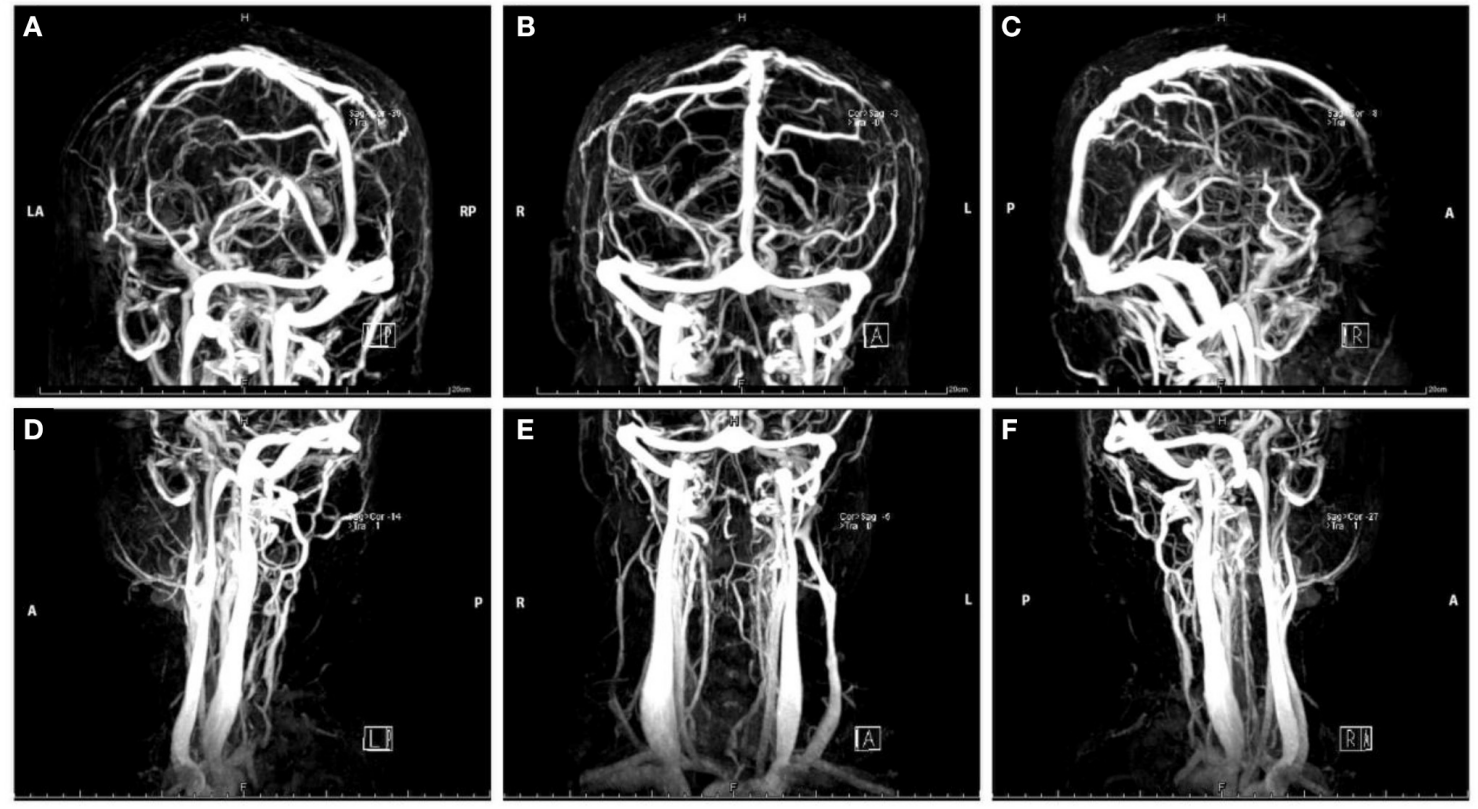
Magnetic resonance venography images of the head **(A–C)** and neck **(D–F)** in Case 2.

Peripheral blood test demonstrated mild normocytic normochromic anemia (hemoglobin, 110 g/L, normal range 120–160 g/L; hematocrit 34.8%, normal range 38.0–50.8%). The evaluation of thrombophilia showed increased levels of fibrinogen (4.11 g/L, normal range 2.0–4.0 g/L), D-dimer (1.4 μg/ml, normal range 2.0–4.0 g/L), AT-III (134%, normal range 80.0–120.0%), and protein C (181%, normal range 65.0–140.0%). All the results of serological tests, including aPL, ANA, ANCA, and complements C3 and C4, were negative. Workups of proinflammatory biomarkers, such as CRP, hs-CRP, and IL-6, were all negative. LPOP was 200 mmH_2_O, and a slightly elevated level of protein (57 mg/dl, normal range 15.0–45.0 mg/dl) and WBC count in CSF was found (5 × 10^6^/L). Serum NSE was more than two times higher than the normal upper limit (36.59 ng/ml, normal range 0.0–17.0 ng/ml). MRI indicated cerebral infarction in the left occipital lobe (Figure 3A) and both sides of the cerebellum (Figure 3B). Magnetic resonance angiography (MRA) showed focal stenosis in the distal branches of the left posterior cerebral artery (PCA) and a partial filling defect in both sides of the superior cerebellar arteries (Figure 4). CE-MRV excluded the possibility of CVT (Figure 5). As this patient has not been identified to have any vascular risk factors, such as diabetes mellitus (DM), hypertension, hyperlipidemia, obesity or smoking history, family history of small vessel disease, or state of hypercoagulability, and the evidence of systemic autoimmune diseases was also negative, we assumed that the cerebral atrial infarction was caused by emboli from cardiac source or thrombosis *in situ* secondary to certain unknown injuries. Then, to further evaluate the potential cause of stroke, transesophageal echocardiography (TEE) was conducted, with negative findings of atrial septal abnormalities [patent foramen ovale (PFO), atrial septal defect (ASD), or atrial septal aneurysm (ASA)]. Based on the patient's medical history of using CsA, the direct or indirect adverse effect of CsA may contribute to the damage in arterial vessel walls, which further initiated the formation of thrombosis *in situ*. The usage of CsA was withdrawn after consultation with the department of hematology due to his relatively well-controlled condition of AA. Aspirin (100 mg, qd) was prescribed at discharge.

MRI follow-up at 6 months post-stroke showed no new-onset parenchymal lesions, and his symptoms were partially relieved and evaluated by PGIC scale (PGIC score = 6).

### Literature Review

We searched PubMed Central (PMC) and EMBASE up to Sep 2019 for publications on CsA-induced thrombotic events and drug-induced CVT. We used Medical subject headings and Emtree headings combining with the following keywords: “cyclosporine-A” and “cerebral venous thrombosis OR cerebral vein thrombosis” and “stroke OR Brain Ischemia OR Brain Infarction OR cerebral infarction OR intracerebral hemorrhage OR intracranial hemorrhage.” We also screened reference lists of included articles for additional relevant studies. Intracranial thrombotic events had to be diagnosed by MRI, conventional angiography, computed tomography (CT) angiography, or at surgery or autopsy. Articles written in languages other than English were only selected if they had an English abstract with sufficient data.

**Figure 6:**
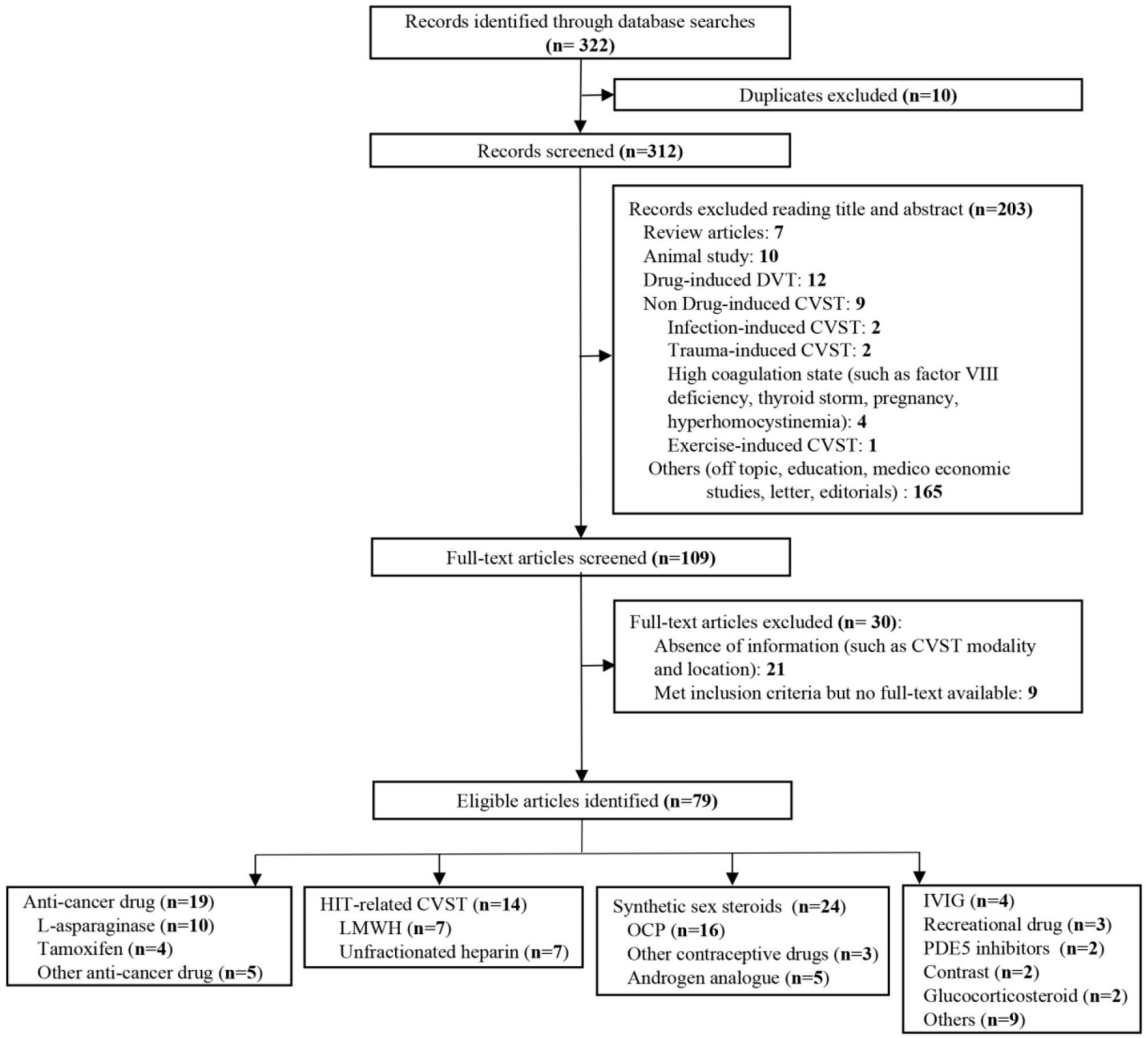
Flow diagram of the study selection process on drug-induced cerebral venous sinus thrombosis (CVST).

We identified 322 publications related to drug-induced cerebral venous sinus thrombosis (CVST), of which 109 were selected for full-length review (Figure 6). Among these, 79 articles with a total of 706 patients were included based on our inclusion criteria. However, nine articles within the inclusion criteria were not collected due to no access to full texts despite that we searched for several times and tried to contact corresponding authors by e-mail. Herein, we listed these nine references in Supplementary Materials. Most of the eligible studies were case reports or case series (*n* = 68) and retrospective studies (*n* = 9), and only one meta-analysis and one prospective study were found (Table 1) (11–89). Western countries reported 95% of the cases, followed by eastern countries (4%), while only one case was from African countries. The mean age of patients was 33.8 ± 17.9 years, and 68.5% of patients were female. There were 94 pediatric cases (94/706, 13.3%). The most common symptoms were seizures (48.6%), headaches (38.1%), nausea/vomiting (19.5%), altered mental status (drowsiness, confusion, syncope, or coma) (17.6%), motor/sensory disorder (12.9%), visual disturbance (9.0%), and aphasia/dysphasia (7.6%). The least common symptoms were personality/behavior change (aggressiveness, *n* = 1; irritability, *n* = 4; poor personal care, *n* = 1) (2.9%) and ataxia (2.4%). Only few cases reported symptoms like general malaise/fatigability (*n* = 2), fever (*n* = 2), diarrhea (*n* = 2), and urinary incontinence (*n* = 1). CVT was confirmed by CE-MRV (*n* = 55) and MRI (*n* = 18). Although digital subtraction angiography (DSA) was considered the gold standard, only 13 cases conducted DSA to make the defined diagnosis. Besides, CT (*n* = 5), CT venography (CTV) (*n* = 5) and autopsy (*n* = 5) were also mentioned as method to detect CVT. Among all sinuses, SSS (*n* = 123) was most likely involved in drug-induced CVT, followed by the TS (*n* = 119), SS (*n* = 97), and straight sinus (*n* = 80). Thrombosis was usually formed bilaterally in the TS (*n* = 26), while it was less common in the left TS (LTS) (*n* = 23) and the right TS (RTS) (*n* = 14). However, the left SS (LSS) more potentially formed thrombosis (*n* = 18) than the right SS (RSS) (*n* = 9); 60.3% of cases had multiple sinus thromboses (105/174). CVST combined with cortical vein thrombosis (CoVT) and isolated CoVT were reported in 102 cases and 6 cases, respectively. Drug-induced deep cerebral vein thrombosis was only found in a vein of Galen, combined with CVST (*n* = 2). Furthermore, CVST was also found to coexist with jugular system thrombosis (*n* = 70), while isolated jugular system thrombosis was very rare (*n* = 2). Nineteen articles indicated contraceptive drug-induced CVT, and 14 studies reported heparin-induced thrombocytopenia (HIT) that resulted in CVT. l-Asparaginase was widely used in patients with acute lymphoblastic leukemia (ALL), while 10 publications demonstrated the close relationship between CVT and l-asparaginase. Furthermore, CsA use was also a risk factor for CVT (*n* = 7).

**Table 1:** Drug induced cerebral venous thrombosis.

**Drug**	**Age/gender**	**Primary disease**	**Symptoms**	**CVST**		**References**	**Country**	**Article type**	**Study size**
				**Modality**	**Location**				
**Contrast**									
Iopamidol	36/F	Myelography	NR	DSA	SSS	(11)	France	Case report	1
	24/M	Recurrent left sciatica (Myelography)	Headache (severe), nausea and vomiting	DSA	SSS, RLS	(12)	France	Case report	1
**Recreational drug**									
MDMA type synthetic drugs									
Ecstasy	22/F	None	Headache, nausea, visual disturbance (photophobia, visual fortification spectra), expressive dysphasia, and right hemisensory loss.	DSA	TS	(13)	UK	Case report	1
Speed	19/F	None	Uncontrollable aggressiveness	MRV	LTS	(14)	Spain	Case report	1
Cocaine	30/M	None	Headache (Occipital) and vomiting	MRV	SSS, TS	(15)	UK	Case report	1
**Phosphodiesterase-5 (PDE5) inhibitors**									
Tadalafil	45/M	None	Headache (severe, posterior, sudden-onset, 3-day), and seizure (generalized tonic-clonic)	MRI	RCoVT	(16)	Japan	Case report	1
Sildenafil	57/M	Two episodes of venous thrombosis (DVT; hemorrhoid plexus thrombosis)	Headache (occipital, 2-week) and visual disturbance (blurry vision)	MRV	SSS, RSS, RTS	(17)	Italy	Case report	1
**IVIG**	16/F	ITP	Headache (severe), neck rigidity, and vomiting	MRI	SSS	(18)	USA	Case report	1
	11/M	ITP	Headache (severe, transient frontotemporal)	CTV	SSS	(19)	Canada	Case report	1
	11/M	Humoral immunodeficiency (Bruton's disease)	Expressive aphasia, right upper extremity heaviness	CTV	SSS	(20)	Lebanon	Case report	1
	13/F	ITP	Headache	MRI	LIJV	(21)	USA	Case report	1
**Others**									
Dulaglutide	52/F	DM-2	Headache (3-day) and visual disturbance (blurry vision)	MRV	RTS	(22)	India	Case report	1
Romiplostim	45/F	ITP	Headache (severe, occipital)	MRV	SSS, TS	(23)	Taiwan	Case report	1
Epoetin alfa	37/F	End stage renal disease	Headache (progressive, several-day)	MRV	SSS, SS	(24)	USA	Case report	1
Dietary supplements	63/M	well-controlled hypertension	Seizure (generalized tonic-clonic)	DSA	SS, vein of Galen	(25)	USA	Case report	1
Lithum	30/F	Bipolar disorder	Headache (progressive), confusion, visual disturbance (blurry vision), and left hemiparesis.	DSA	SSS, LSS, LTS, straight sinus	(26)	USA	Case report	1
Finasteride	35/M (case 1) 41/M (case 2)	Male-pattern hair loss	Headache and seizures (case 1); Headache (case 2)	CTV	SSS	(27)	Japan	Case report	2
Combination tacrolimus/sirolimus	67/M	Renal transplantation	Seizure (generalized) and right hemiparesis	MRI	TS	(28)	Australia	Case report	1
Clozapine	30/F	Chronic paranoid schizophrenia	Vomiting, irritability, fatigability, poor personal care (5-day)	MRV	SSS, ISS, RTS, RIJV	(29)	USA	Case report	1
Levetiracetam	6.5/M	None	Headache and vomiting (2-day), then seizures (generalized)	MRI	LTS, LSS	(30)	UK	Case report	1
**Synthetic sex steroids**									
**Oral contraception**									
Phytoestrogens	52/F	None	Headache (diffuse and continuous, 2-month)	MRV	LLS, LSS	(31)	Portugal	Case report	1
Third-generation CHCs (containing desogestrel or gestoden)	16–49/F	CVT^h^	Seizure (*n* = 52)	CTV/MRV	CVT: dural sinuses, DCVT, CoVT, IJV	(32)	USA	Retro	57
Ethinylestradiol/ levonorgestrel	21/F	None	Headache (severe, 4-day)	CTV	SSS, CoVT, RTS, RSS	(33)	USA	Case report	1
NR	18/F (case 1) 23/F (case 2)	Protein C resistance (case 1); Anti-thrombin III deficiency (case 2)	Headache, nausea, vomiting and visual disturbance (photophobia) (case 1); Headache, vomiting, altered sensorium (case 2)	MRI	SSS, TS, LSS (case 1) SSS (case 2)	(34)	India	Case report	2
NR	18/F (case 1) 18/F (case 2)	None (case 1); ADHD and bipolar disorder (case 2)	Headache (intermittent right-sided) and visual disturbance (blurry vision) (3-day) (case 1); Headache (intermittent right-sided) and visual disturbance (double vision) (6-week) (case 2)	MRV	LTS, LSS (case 1); RTS, IJV (case 2)	(35)	USA	Case report	2
NR	27/F (case 1) 23/F (case 2)	None	Headache (retroauricular), vomiting, drowsiness, fever (several days) (case 1); Headache, vomiting, increasing drowsiness and extra-pyramidal movements (1 day) (case 2).	DSA (case 1) MRI (case 2)	LTS, SS (case 1); SSS, SS (case 2)	(36)	Italy	Case report	2
Ethinylestradiol/ desogestrel	24/F	None	Headache (severe, 1 week), vomiting.	DSA	SSS, RLS, RSS, vein of Galen	(37)	Czech Republic	Case report	1
Noracyclin^a^ (case 1) Ovulen^b^ (case 2)	50/F (case 1) 41/F (case 2)	None (case 1); Thrombosis of left common carotid (case 2)	Fluctuated conscious status, aphasia, right arm weakness, and several epileptic seizures (case 1); Right hand numbness and rapid-onset unconsciousness (case 2)	Necropsy	SSS, CoVT (case 1, 2);	(38)	Switzerland	Case report	2
Lyndiol 2, 5 (case 1) Metrulen-M (case 2) Anovlar (case 3) Gynovlar (case 4) Nuvacon (case 5)	24/F (case 1) 49/F (case 2) 30/F (case 3) 23/F (case 4) 29/F (case 5)	RIJVS (case 1) Diabetes (case 2) Right pulmonary embolus (case 3) Thrombosis of choroid plexus (case 4) Marfan's syndrome; thrombosis of iliac vein (case 5)	Headache, vomiting and drowsiness (5-day) (case 1); Headache, vomiting, seizure (generalized, left-sided) (case 2); Deeply comatose (case 3); Diarrhea, headache (severe) and further unconsciousness (case 4); Abdominal pain, headache (severe, 1-day), and vomiting (5-day) (case 5).	Necropsy	SSS, RTS, SS, CoVT, IJV (case 1); SSS, RTS, LTS, CoVT (case 2, 3, 5); All sinuses thrombosis, CoVT (case 4)	(39)	NR	Case report	5
Norethynodrel and mestranol	35/F	Eclampsia during pregnancy; obesity	Headache (severe, persistent, temporal) (4-day), vomiting, diarrhea, seizure, urinary incontinence, upper limb weakness (left-sided), and visual disturbance (photophobia)	Necropsy	SSS, LTS, CoVT	(40)	NR	Case report	1
Enovid (case 1) Ortho novum (case 2)	29/F (case 1, 2)	Multiple arterial thrombi; thrombosis of left opththalmic vein; Marfan's syndrome (case 2)	NR	Necropsy	All sinuses thrombosis, CoVT (case 1); SSS (case 2)	(41)	NR	Case report	2
Combined oral contraceptives (COCs), progestin-only contraceptives or cyproterone acetate.	15–49/F	Former thromboembolic event (including PE, CVT, ischemic stroke, or MI)	NR	NR	CVT	(42)	France	Retro	452
Yasmin 28^c^	18/F	None	Headache (Throbbing, frontal and occipital, 1-month)	MRV	RTS, RSS, IJV, CoVT	(43)	USA	Case report	1
NR	22/F	None	Severe headache (1 week)	CT/MRV/DSA	LTS, LSS	(44)	China	Case report	1
Cyproterone/gestodene	50/F	Heterozygous factor II polymorphism	NR	NR	CVST	(45)	Italy	Retro	1/28
NR	23–45/F	Prothrombin mutation G20210A (*n* = 2)	Headache (*n* = 11), vomiting (*n* = 2), aphasia (*n* = 1)	MRA (*n* = 10) DSA (*n* = 7)	Straight sinus (*n* = 15) TS (*n* = 7)	(46)	Spain	Retro	15
**Other contraceptive drugs**									
Norethisterone enanthate injection	23/F	None	Headaches (progressive), vomiting (repeated) and syncope (2–3 min)	MRV	SSS, RTS, RSS	(47)	USA	Case report	1
Vaginal contraceptive ring^d^	28/F	None	Headache	CT	LSS, TS	(48)	USA	Case report	1
	33/F	None	Seizures (multiple tonic-clonic), headaches	CT	LTS, LSS, LIJV	(49)	Canada	Case report	1
**Androgen analog**									
Oxymetholone	40/F	AA	NR	MRI	SSS, LTS	(50)	South Korea	Case report	1
Nandrolone decaonoate	22/M	None	Headaches and vomiting (repeated) (3-day)	MRV	SSS, TS	(51)	Iran	Case report	1
Fluoxymesterone	52/F (case 1) 39/F (case 2)	Hypoplastic anemia	Headaches (severe), seizures, aphasia and hemiplegia, coma (case 1) Headaches (severe) and seizure (focal) (case 2)	DSA	SSS (case 1) SSS, CoVT (case 2)	(52)	USA	Case report	3
Methenolone-enanthate	26/F (case 3)		Headaches, visual disturbance (blurred vision), and hemiparesis (right-sided) (case 3)		SSS, CoVT (case 3)				
Danazol^e^	40/M	AA	Headache (acute onset) and altered sensorium	CT	CoVT	(53)	India	Case report	1
	19/M	IHA	Headache and visual disturbance (transient obscurations of vision)	DSA	SSS, CoVT, straight sinus	(54)	USA	Case report	1
**Steroid**	32/F	Relapsing-remitting multiple sclerosis	Numbness and weakness (both legs)	MRV	LTS, LSS	(55)	Turkey	Case report	1
	31/M	IHA	Headaches, anorexia, general malaise	DSA	SSS, CoVT, straight sinus	(52)	Japan	Case report	1
**HIT-related CVST**									
LMWH	60/F	Bilateral extensive varicose veins in legs	Right focal seizures with secondary generalization followed by headache, slurred speech, and altered sensorium	MRI/CTV	LTS, LSS	(56)	India	Case report	1
	52/F	Kyphoplasty and posterior spinal fusion	Acute onset altered mental status with significant agitation and non-sensical speech.	MRV	LTS, LSS, LIJV	(57)	USA	Case report	1
	55/F	Partial gastrectomy	NR	MRV	SSS, SS, LIJV	(58)	Germany	Case report	1
	57/F	Antiphospholipid syndrome and possible systemic lupus erythematosus	Fever, altered mental status, and aphasia (expressive and sensory)	MRV	SSS, LSS, LIJV	(59)	Greece	Case report	1
	69/F	Knee replacement for osteoarthritis	Seizures (right arm focal type)	MRV	SSS, CoVT	(60)	USA	Case report	1
	72/M	Left knee joint surgery	Comatose	Necropsy	SSS, SS, CoVT	(61)	Germany	Case report	1
	38 ± 28	NR	NR	MRV	CVT	(62)	Germany	Retro	3/120
Unfractionated heparin	61/F	Retinal transient ischemic attack; DVT of the leg	Headache (progressive) and aphasia	MRI	LLS	(63)	France	Case report	1
	18/M	Extensive UC	Headache (severe), nausea and vomiting	MRI	RTS, confluence area	(64)	Sweden	Case report	1
	45/F	Cystic pituitary adenoma.	Aphasia and visual disturbances	MRI	LTS, LSS	(65)	USA	Case report	1
	67/F	NR	NR	CTV	SSS	(66)	Germany	Case report	1
	63/F	Polycythemia vera	Seizures (right-sided focal type)	Contrast-enhanced CT	SSS	(67)	USA	Case report	1
	36/F	PNH	Headache, nausea, then developed dysphasia and right hemiparesis	MRV	LTS, LSS	(68)	Japan	Case report	1
	67/F	Antiphospholipid syndrome	Headache (transient), vertigo, tinnitus and right hemifacial par-aesthesia with propagation down to the ipsilateral arm.	MRV	RTS, RSS, IJV	(69)	Switzerland	Case report	1
**Anti-cancer drugs**									
Tamoxifen	40/F	Breast cancer	Headache and hemiparesis (left-sided) (10-day)	MRI	SSS, RLS, RIJV	(70)	Turkey	Case report	1
	30/F	Breast cancer	Headache (acute-onset) and hemiparesis (left-sided)	DSA/MRI	SSS	(71)	South Korea	Case report	1
	46/F	Breast cancer	Headache (severe) and nausea (subacute onset, 2-week)	MRI/CT	SSS, straight sinus	(72)	South Korea	Case report	1
	47/F	Breast cancer	Headache (Severe), seizure (generalized tonic-clonic)	MRV	SSS, CoVT	(73)	USA	Case report	1
L-asparaginase	15/M	ALL	Acute severe headache and recurrent vomiting	MRV	SSS, TS, straight sinus	(74)	Germany	Case report	1
	10/M (case 1) 13/F (case 2)	ALL (case 1) Acute mixed phenotypic leukemia (case 2)	Headache, vomiting, seizures and loss of consciousness (case 1) Headache and focal seizure (case 2)	MRV	SSS (case 1 & 2)	(75)	India	Case report	2
	5–16	ALL (*n* = 8) Non-Hodgkin lymphoma (*n* = 1)	Headaches (chronic, daily), and seizures (partial-complex)^g^	MRV	LTS, LSS, LIJV (case 1) CVST (case 2) SSS (case 3) TS (case 4)	(76)	USA	Retro	9/200
	2.3/M (case 1) 3.5/F (case 2)	ALL (case 1 & 2)	Seizure (left focal seizure evolving into generalized tonic-colonic seizure and subsequently status epilepticus) (case 1); Seizure (left focal seizure evolving into status epilepticus) (case 2)	MRV (case 1 & 2)	SSS (case 1 & 2)	(77)	India	Case report	2
	(1–17)/(38/10, M/F)	ALL	Headache (*n* = 14), a decreased level or loss of consciousness (*n* = 15), visual impairment (*n* = 3), focal or generalized seizures (*n* = 18), photophobia (*n* = 1), vomiting (*n* = 8), irritability (*n* = 3), hemiparesis (*n* = 5), ataxia (*n* = 2), speech impairment (*n* = 6), and cranial nerve palsy (*n* = 1).	CT (*n* = 38), MRV (*n* = 27)	CoVT (*n* = 3), CVST (*n* = 26), CVST combined with CoVT (*n* = 4)	(78)	Italy	Retro	33/48
	5.6 (1.0–17.0)/(38/33, M/F)	ALL	NR	MRI	CVT	(79)	Austria	Pro	3/71
	9/M	ALL	Headache (Acute-onset, severe) and then seizures (left-sided focal type) and right arm sensory disturbance.	MRI	SSS	(80)	Saudi Arabia	Case report	1
	32 (15–59)/(144/96, M/F)	ALL or lymphoblastic lymphoma	NR	NR	CVT	(81)	France	Retro	5/214
	NA	ALL	NR	NR	CVT	(82)	Italy	Meta-analysis	26/1,752
	16/M	ALL	Headache, vomiting, and multiple episodes of seizures	Contrast enhanced CT	CoVT	(83)	India	Case report	1
L-asparaginase or Tamoxifen^f^	44.5 (10-71)/(16/4, M/F)	Hematologic malignancies (*n* = 9); Solid tumor (*n* = 11)	Headache (*n* = 8), seizure (*n* = 6), nausea/vomiting (*n* = 5), hemiparesis/aphasia (*n* = 4), altered metal status/coma (*n* = 3), dizziness (*n* = 3), visual disturbance (*n* = 2), gait disturbance (*n* = 1), incidental finding (*n* = 1), not available (*n* = 1)	MRV	SSS (*n* = 13), TS (*n* = 8), SS (*n* = 5), IJV (*n* = 4), straight sinus (*n* = 1)	(84)	USA	Retro	20
Cisplatin and BEP	16/F	Immature teratoma	Hemiparesis (left-sided)	MRI	SSS	(85)	Tunisia	Case report	1
Thalidomide	74/F	Multiple myeloma	Headache (right-sided, frontal), confusion and speech difficulty (acute-onset)	MRI	LTS, straight sinus, CoVT, LIJV	(86)	USA	Case report	1
Methotrexate	12/M	ALL	Hemiparesis (right-sided), aphasia, altered mental status, persistent seizure activity, and progressive neurological deterioration	MRI	LTS, LSS	(87)	USA	Case report	1
ATRA	22/F	APML	Visual disturbance (blurred vision on the right eye)	MRV	SSS, RSS, TS, IJV	(88)	Malaysia	Case report	1

*R, right; L, left; SS, sigmoid sinus; SSS, superior sagittal sinus; TS, transverse sinus; LS, lateral sinus; CVT, cerebral venous thrombosis; CVST, cerebral venous sinus thrombosis; CoVT, cortical vein thrombosis; IJVS, internal jugular vein stenosis; DVT, deep vein thrombosis; ALL, acute lymphoblastic leukemia; APML, acute promyelocytic leaukaemia; HIT, Heparin-induced thrombocytopenia; AA, Aplastic anemia; DM, diabetes mellites; PNH, Paroxysmal nocturnal hemoglobinuria; UC, ulcerative colitis; MI, myocardial infarction; ADHD, attention deficit hyperactivity disorder; IHA, immune hemolytic anemia; ITP, immune thrombocytopenia; PE, pulmonary emboli; MRI, magnetic resonance imaging; CTV, CT venography; MRV, magnetic resonance venography; DSA, digital subtraction angiography; CHCs, combined hormonal contraceptives; ATRA, All-trans retinoic acid; BEP, bleomycin and etoposide; IVIG, Intravenous immunoglobulins; Retro, retrospective; Pro, prospective; NR, not reported*.

a *Noracyclin = Lynestrenol/mestranol*.

b *Ovulen = Ethinodiol diacetate/mestranol*.

c *Yasmin 28 = drospirenone*.

d *Vaginal contraceptive ring (NuvaRing): etonogestrel and ethinyl-estradiol per day*.

e *Danazol is an attenuated androgen derived from ethisterone (17 α-ethinyltestosterone)*.

f *This study enrolled patients with hematologic malignancies or solid tumors*.

g *All patients survived, with 4 experiencing complications possibly related to CVST. In this article, report these four patients in detail*.

h *Female patients with diagnosed cerebral venous thrombosis (CVT) of the dural sinuses, involvement of the deep venous system (DCVT), cortical venous thrombosis, or with thrombosis of the jugular system*.

**Figure 7:**
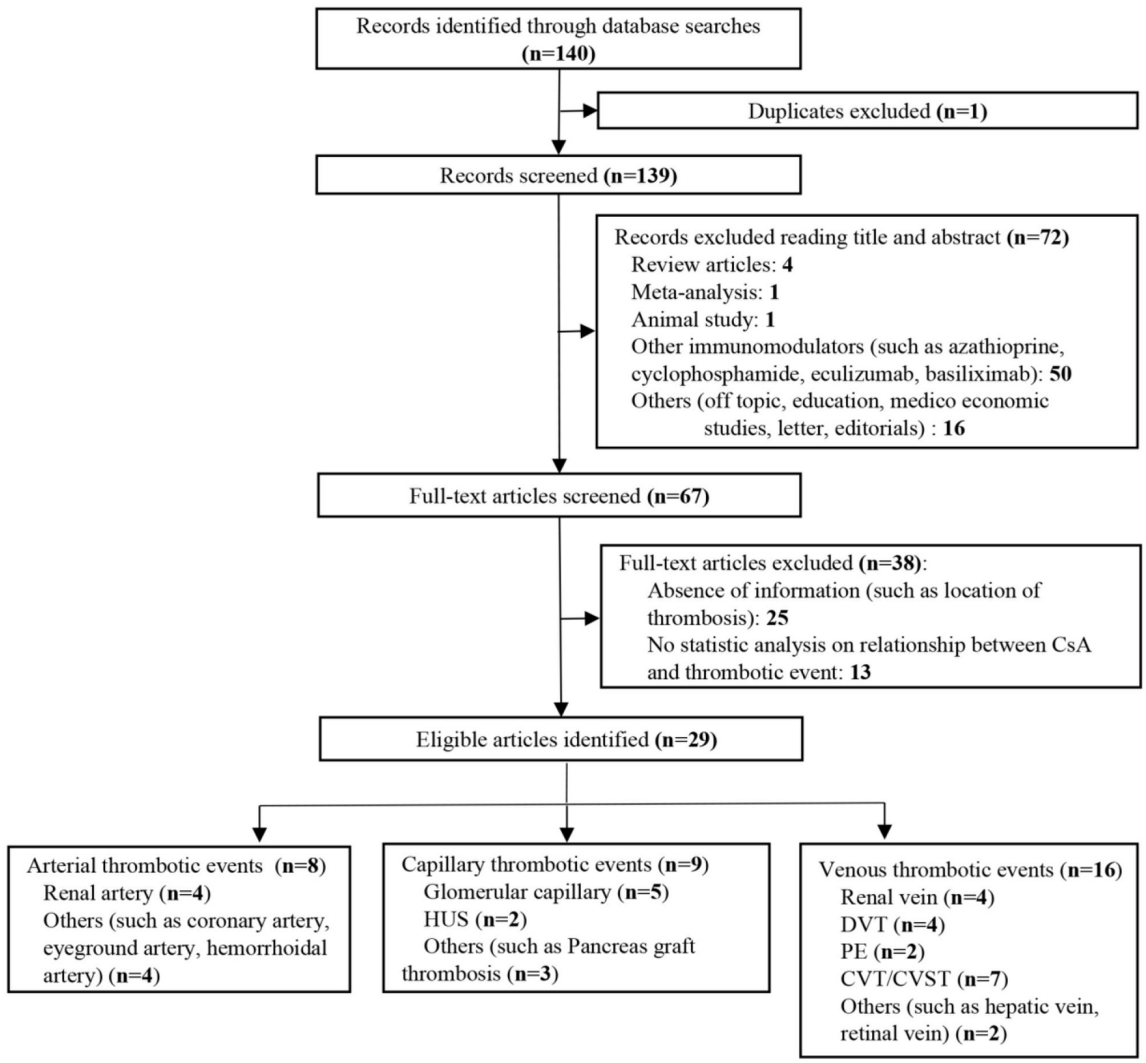
Flow diagram of the study selection process on cyclosporine-A (CsA)-induced thrombosis.

We further searched articles related to CsA-induced thrombotic events to explore if CsA would bring extensive damage to different kinds of blood vessels. One hundred forty articles were identified, and full texts of 67 articles were screened (Figure 7). Only studies with sufficient information and a clear description of the relationship between CsA and thrombosis were finally included (*n* = 29). CsA was more likely associated with venous thrombotic events (*n* = 16), followed by capillary thrombotic events (*n* = 9) and arterial thrombotic events (*n* = 8). CVT was the most common thrombosis in CsA-induced thrombotic events (Table 2) (1–9, 90–109). Thrombosis in the renal vessel system was more likely formed due to CsA use in renal transplantation (*n* = 13).

**Table 2:** Cyclosporine-A induced thrombosis.

**Age/gender**	**Thrombosis location**	**Primary disease**	**References**	**Country**	**Study size**	**Article type**
NR	DVT (*n* = 25), PE (*n* = 4), DVT with PE (*n* = 11)	Renal transplantation	(90)	UK	40/480	Retro
41 ± 12	DVT	Renal transplantation	(91)	Switzerland	9/97	Pro
52/M (case 1) 26/M (case 2) 32/M (case 3) 61/M (case 4) 11/M (case 5) 54/M (case 6) 45/F (case 7)	Glomerular capillary; Renal afferent artery	Renal transplantation (cadaver-donor)	(92)	UK	7	Case report
NA	Cyclosporine-associated arteriopathy (acute tubular necrosis; acute vasculitis; glomerular ischemia; interstitial intimate; Intima proliferation; venous thrombosis)	Renal transplantation (cadaver-donor)	(93)	USA	16/200	Retro
36.7 ± 1.3/(49/41, M/F)	PE (*n* = 10), Renal vein (*n* = 1), DVT (*n* = 3), Hemorrhoidal artery (*n* = 3)	Renal transplantation (cadaver-donor)	(8)	Belgium	13/90	Retro
35.9 ± 13.8	DVT	Renal transplantation (cadaver-donor, living-donor)	(94)	Sweden	9/97	Pro
53/F (case 1) 33/M (case 2) 48/F (case 3)	HUS, Glomerular capillaries, Renal artery	Renal transplantation	(95)	Canada	3	Case report
33/F	Glomerular capillaries	Renal transplantation (cadaver-donor)	(96)	UK	1	Case report
NA	Glomerular capillaries (Platelet microthrombi)	Renal transplantation	(97)	UK	12/32	Retro
NA	Renal artery	Renal transplantation (cadaver-donor, living-donor)	(98)	China	1/14	Retro
6 (case 1) 6 (case 2) 17 (case 3) 48 (case 4) 53 (case 5) 51 (case 6)	Renal vein	Renal transplantation (cadaver-donor, living-donor)	(99)	UK	6/791	Retro
NA	HUS, Glomerular capillaries	Behcet's disease	(100)	France	2	Case report
NA	CVST	SSINS	(101)	UK	1/53	Retro
45.1 ± 12.3	Renal vein	Renal transplantation (cadaver-donor)	(102)	UK	16	Pro
NA	Eyeground artery	Recurrent nephrotic syndrome	(103)	Japan	1	Case report
NA	CVT	Renal transplantation	(104)	Spain	1	Case report
25.0 ± 26.4	HUS	Renal transplantation	(105)	USA	10/672	Retro
NA	Hepatic vein	Inflammatory bowel disease and latent thrombocythemia	(106)	France	1	Case report
19/M	CVST	Severe active UC	(5)	Japan	1	Case report
48/F	CVST (LTS, LSS), LIJVS	Chronic UC	(4)	USA	1	Case report
NA	Thrombotic microangiopathy	SPK transplantation	(107)	Belgium	1/102	Retro
(18–55)	Pancreas graft thrombosis (*n* = 10), Kidney graft thrombosis (*n* = 1)	SPK transplantation	(9)	Belgium	11/102	Pro
31 ± 11	Graft thrombosis (combined arterial and venous thrombotic occlusion, *n* = 5; arterial occlusion, *n* = 3, venous occlusion, *n* = 1)	SPK transplantation	(3)	Austria	9/67	Retro
30/F	Central retinal vein	Renal transplantation (cadaver-donor)	(108)	Croatia	1	Case report
56.7 ± 10.1	Coronary artery	Heart transplantation	(2)	Canada	18/129	Retro
25/M	CVST (SSS, TS)	Renal transplantation (living-donor)	(1)	Sri Lanka	1	Case report
23/F	CVT	Neuro-Behcet's disease	(109)	Brazil	3/40	Retro
18–64/(28/33, F/M)	Venous thrombosis	Acute steroid-refractory or dependent UC	(6)	Finland	1/61	Pro
44/F	CVST	AA	(7)	China	1	Case report

*R, right; L, left; CVT, cerebral vein thrombosis; CVST, cerebral vein sinus thrombosis; CoVT, cortical vein thrombosis; SSS, superior sagittal sinus; TS, transverse sinus; DVT, deep vein thrombosis; PE, Pulmonary emboli; SPK transplantation, Simultaneous pancreas-kidney transplantation; HUS, Hemolytic Uremic Syndrome; AA, Aplastic anemia; SSINS, steroid sensitive idiopathic nephrotic syndrome; UC, ulcerative colitis; IBD, inflammatory bowel disease; Retro, retrospective; Pro, prospective; NR, not reported*.

### Statistical Analysis

Quantitative variables with a normal distribution were specified as mean ± standard deviation. Analyses were performed with Stata software (version 15.0 SE, Stata Corp, LP, Texas, USA).

## Discussion

This was the first systematic review on drug-induced CVT and CsA-related thrombosis based on the clinical cases. CVT is a rare subtype of stroke, accounting for <1% of all strokes (110). Severe CVT can be fatal. Common etiologies of CVT are postpartum period, infection, and coagulopathies (111). However, drug-induced CVT should not be neglected, as this kind of CVT could be reversible and preventable if we avoid certain drugs when treating primary diseases, for instance, the two cases presented in this study. In line with CVT of other etiologies, the most common symptoms in drug-induced CVT were seizures (48.6%) and headaches (38.1%). Furthermore, women or young people were mainly involved. Both CE-MRV and black-blood thrombus image (BBTI) are useful imaging tools to make a definitive diagnosis.

It would be worth noticing that CsA can induce not only CVT but also cerebral arterial thrombosis, as in Case 2 of this report. Interestingly, drug-induced CVT is more likely involved in multiple sinuses, cortical veins, or IJV, such as Case 1 in this paper. It is well-known that OCP can promote CVT in women, whereas CsA-related CVT should also raise our concern.

Cyclosporine thrombogenicity manifested mostly with CVT. However, the underlying mechanism is still controversial. Several adverse effects of CsA had been reported in patients: Firstly, CsA enhanced secretion of von Willebrand factor (VWF), a classic platelet agonist, from endothelial cells (112). Then, platelet aggregation was increased due to a higher level of VWF in circulation (113). Thirdly, CsA-induced endothelial cell dysfunction by suppressing nitric oxide production and initiating intrinsic coagulation pathway (10, 114). Further, CsA was associated with increased D-dimer and fibrinogen levels, which were observed in our patients after the onset of the thrombotic event, which was consistent with other studies (4, 8, 115). However, some animal and clinical studies showed that CsA therapy was not related to thrombosis in renal transplant and even provided strong protection from both reperfusion injury (97) and congestive heart failure (116) or improved recovery after treatment of coronary thrombosis with angioplasty (117).

Moreover, apart from the thrombogenic effect of CsA, patients with AA frequently presented with decreased levels of WBC, RBC, or platelet. Anemia secondary to AA could also be associated with both CVT (118) and arterial ischemic stroke (AIS) (119). More importantly, anemia was correlated with stroke severity and poor clinical outcomes in AIS patients (120, 121). Thus, a well-controlled condition of AA is vital to prevent cerebral thrombotic events. Besides, a stronger association between anemia and CVT in men than in women (118), which reminded us that the potential confounders, such as age and gender, should also be taken into consideration when treating AA patients with thrombotic complications.

Although we cannot prove the clear relationship between the potential adverse effect of CsA, anemia secondary to AA, and intracranial thrombotic events in these two cases due to the rarity of similar cases, CsA-induced intracranial thrombosis in AA patients was firstly reported. This observation may at least warrant caution of monitoring thrombotic events during CsA treatment in patients with AA. Therefore, we suggested that future studies could shed more light on the mechanism of the prothrombotic effects of Cs-A in the treatment of AA patients. Additionally, the systematic literature review on CsA-related thrombotic events and drug-induced CVT would give more clinical references to physicians in this field, especially when treating patients with unknown reasons for stroke.

## Summary Table

### What Is Known About This Topic?

- A possible association may exist between cyclosporine-A use and thrombotic events in patients with aplastic anemia.
- Currently, there is a lack of information on comprehensive review on drug-induced cerebral venous thrombosis and cyclosporine-A-related thrombotic events.

### What Does This Paper Add?

- This real-world study provides two cases with aplastic anemia that developed intracerebral thrombotic events due to cyclosporine-A use.
- Articles on cyclosporine-A-related thrombotic events were reviewed. CsA-induced thrombosis may involve the arteries, veins, and capillaries. Damage to the renal vascular system was most commonly reported due to the acute and chronic nephrotoxicity of CsA.
- Studies on drug-induced cerebral venous thrombosis were selected, of which we summarized features of clinical characteristics and neuroimaging findings.

## Data Availability Statement

The original contributions presented in the study are included in the article/Supplementary Material, further inquiries can be directed to the corresponding author.

## Ethics Statement

The studies involving human participants were reviewed and approved by Xuanwu Hospital, Beijing, China. The patients/participants provided their written informed consent to participate in this study. Written informed consent was obtained from the individual(s) for the publication of any potentially identifiable images or data included in this article.

## Author Contributions

RM drafted and revised the manuscript and provided the study concept and design. S-YS drafted and revised the manuscript, provided the study concept and design, and carried out collection, assembly, and interpretation of the data. RM, S-YS, Y-CD, Z-AW, and X-MJ wrote the manuscript and gave final approval of the manuscript. Y-CD intensively edited the revised version and contributed to the critical revision. All authors contributed to the article and approved the submitted version.

## Conflict of Interest

The authors declare that the research was conducted in the absence of any commercial or financial relationships that could be construed as a potential conflict of interest.

## Supplementary Material

Supplementary Figure 1 Funduscopic imaging of Case 1.

Supplementary Table 1 Follow up of abnormal examination in Case 1.
