# Supplementary figures and images for: Cyclosporine-A-Induced Intracranial Thrombotic Complications: Systematic Review and Cases Report
Source: Front Neurol. 2021 Feb 11;11:563037. doi: 10.3389/fneur.2020.563037 (PMC7906016; doi:10.3389/fneur.2020.563037)

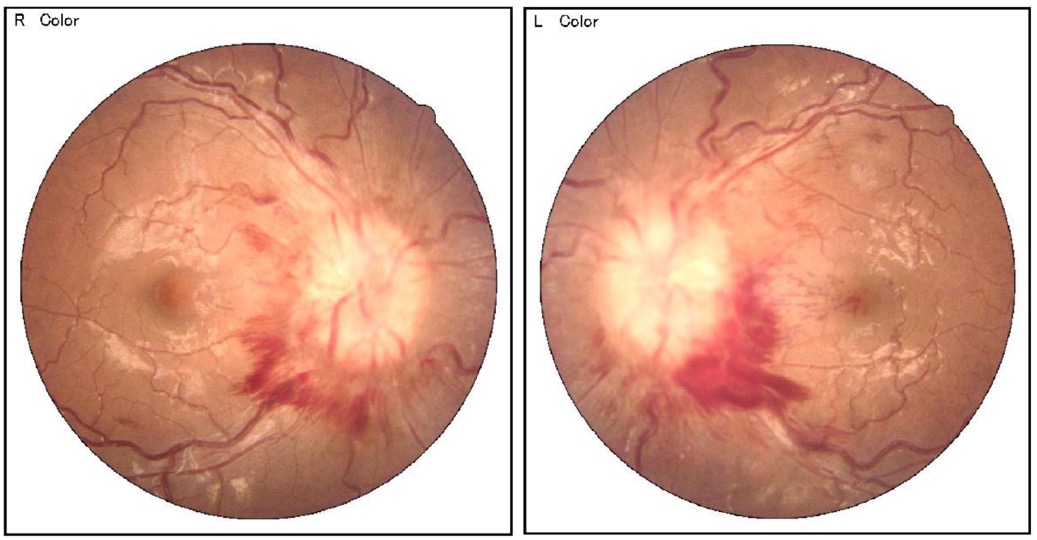

Supplement: Supplementary Figure 1 — Funduscopic imaging of Case 1. [file Image_1.JPEG]
