# Supplementary material for: Cyclosporine-A-Induced Intracranial Thrombotic Complications: Systematic Review and Cases Report
Source: Front Neurol. 2021 Feb 11;11:563037. doi: 10.3389/fneur.2020.563037 (PMC7906016; doi:10.3389/fneur.2020.563037)
Supplement: Supplementary Table 1 — Follow up of abnormal examination in Case 1. [file Data_Sheet_1.DOCX]

Supplementary materials

The following references are studies eligible for our inclusion criteria, however, we couldn’t find full text of them. We list them based on publication date [1-9]:

1. Shinohara, K., et al., *Oxymetholone treatment in aplastic anemia.* Nihon Ketsueki Gakkai Zasshi, 1974. **37**(3): p. 255-65.

2. Galimberti, J., et al., *Clinical and hematogenic considerations on 2 cases of cerebral venous thrombosis occuring during the use of oral estro-progestational preparations.* Annali dell'Ospedale Maria Vittoria di Torino, 1976. **19**(7-12): p. 183-193.

3. Baldini, M. and G. Sangiovanni, *Benign intracranial hypertension and thrombosis of the venous sinuses caused by contraceptive treatment. Anatomo-clinical and neuroradiological observations.* Giornale italiano di chemioterapia, 1977. **24**(1-2): p. 117.

4. Rancurel, G., et al., *Synthetic estro-progestational (contraceptive) agents and cerebral ischemic complications: cerebral pseudo-tumours, thrombosis of intracranial venous sinuses. Apropos of 60 cases.* La semaine des hôpitaux : organe fondé par l'Association d'enseignement médical des hôpitaux de Paris, 1980. **56**(39-40): p. 1583-1587.

5. Hitosugi, M., et al., *[A case of dural sinus thrombosis during the medication of medroxyprogesterone acetate].* Nihon Hoigaku Zasshi, 1997. **51**(6): p. 452-6.

6. Hill, L.W. and F.M. Hindelang, *Cerebral venous thrombosis as a complication of oral contraceptive use in a 15-year-old patient: a case report.* The Journal of the Louisiana State Medical Society : official organ of the Louisiana State Medical Society, 2007. **159**(4): p. 195, 197.

7. Mogensen, S.S., A. Abild-Nielsen, and N.H. Bjarnason, *[Sinus thrombosis in a healthy female taking oral contraceptives].* Ugeskrift for laeger, 2011. **173**(39): p. 2422-2423.

8. Sikorska, A., et al., *Cerebral venous and sinus thrombosis complicated by heparin-induced thrombocytopenia.* Hematologia, 2011. **2**(4): p. 363-369.

9. Ishihara, M., et al., *Cerebral venous sinus thrombosis following cisplatin-based chemotherapy for testicular tumor.* Neurological Surgery, 2017. **45**(5): p. 417-422.

Supplementary table 1. Follow up of abnormal examination in **Case 1.**

| Hospital Day | Day 1 | Day 5 | Day 6 | Day 7 | Day 8 | Day 9 | Day 10 | Day 11 | Day 12 | Day 13 | Day 14 | Day 15 | Day 16 | Day 17 | Day 18 | Day 19 |
| --- | --- | --- | --- | --- | --- | --- | --- | --- | --- | --- | --- | --- | --- | --- | --- | --- |
| WBC (*10^9/L) | 3.7 | 4.35 | 5.40 | 5.52 | 3.9 | 3.57 | 4.66 | 5.51 | 4.96 | 4.62 | 3.98↓ | 3.89↓ | 2.75↓ | 2.63↓ | 3.72↓ | 4.50 |
| RBC (*10^12/L) | 2.8↓ | 2.58↓ | 2.54↓ | 2.33↓ | 2.32↓ | 2.49↓ | 2.46↓ | 2.50↓ | 2.37↓ | 2.43↓ | 2.35↓ | 2.15↓ | 1.93↓ | 1.97↓ | 1.92↓ | 2.03↓ |
| Hb (g/L) | 85↓ | 80↓ | 80↓ | 72↓ | 72↓ | 77↓ | 81↓ | 77↓ | 72↓ | 75↓ | 72↓ | 67↓ | 62↓ | 61↓ | 60↓ | 64↓ |
| MCV (fL) | 95.4 | 94.2 | 98.4 | 94.8 | 95.3 | 95.6 | 94.3 | 94.8 | 94.5 | 94.7 | 94.9 | 94.4 | 94.3 | 95.4 | 96.4↑ | 96.1↑ |
| Hct (%) | 26.7↓ | 24.3↓ | 25↓ | 22.1↓ | 22.1↓ | 23.8↓ | 24.9↓ | 23.7↓ | 22.4↓ | 23.0↓ | 22.3↓ | 20.3↓ | 18.2↓ | 18.0↓ | 18.5↓ | 19.6↓ |
| RDW (%) | 16.6↑ | 17.0↑ | 17.8↑ | 17.4↑ | 17.4↑ | 17.3↑ | 17.0↑ | 17.5↑ | 17.7↑ | 23.0↑ | 17.6↑ | 17.7↑ | 18.0↑ | 18.4↑ | 18.6↑ | 18.8↑ |
| PLT (*10^9/L) | 91↓ | 71↓ | 80↓ | 68↓ | 66↓ | 79↓ | 75↓ | 67↓ | 58↓ | 59↓ | 63↓ | 56↓ | 52↓ | 63↓ | 60↓ | 64↓ |
| Fibrinogen (g/L) | 4.21↑ | 3.73 | 4.24↑ | 3.67 | 3.43 | 3.80 | 3.92 | 4.79↑ | 4.74↑ | 5.9↑ | 5.97↑ | 4.68↑ | 4.22↑ | 3.92↑ | 4.28↑ | 5.06↑ |
| D-dimer (μg/mL) | 2.47↑ | 9.76↑ | 9.19↑ | 5.48↑ | 2.24↑ | 1.67↑ | 1.63↑ | 6.73↑ | 12.13↑ | -- | -- | -- | -- | -- | -- | -- |

Normal range: WBC 4.0-10.0 10^9/L, RBC 3.5-5.5*10^12/L, Hb 110.0-150.0 g/L, MCV 82.0-95.0 fL, Hct 33.5-45.0%, RDW 0.0-15.0%, PLT 100.0-300.0*10^9/L, Fibrinogen 2.0-4.0 g/L, D-dimer 0.01-0.5 μg/mL
